# Von Hippel–Lindau Disease (VHL): Characteristic Lesions with Classic Imaging Findings

**DOI:** 10.15586/jkcvhl.v10i3.293

**Published:** 2023-08-03

**Authors:** Suryansh Bajaj, Darshan Gandhi, Divya Nayar, Ali Serhal

**Affiliations:** 1 Department of Radiology, University of Arkansas for Medical Sciences, Little Rock, AR, USA;; 2 Department of Diagnostic Radiology, University of Tennessee Health Science Center, Memphis, TN, USA;; 3 Department of Neurology, University of Arkansas for Medical Sciences, Little Rock, AR, USA;; 4 Department of Musculoskeletal Radiology, Northwestern Memorial Hospital, Northwestern University Feinberg School of Medicine, Chicago, IL, USA

**Keywords:** carcinoma, cysts, hemangioblastoma, insulinoma, renal cell, Von Hippel–Lindau disease

## Abstract

Von Hippel–Lindau disease (VHL) is a multisystem cancer syndrome caused by the inactivation of the VHL tumor suppressor gene and involves various organ systems including the central nervous system (CNS), endocrine system, and the kidneys. Tumors seen in patients with VHL disease can be benign or malignant and are usually multifocal, bilateral, and hypervascular in nature. As most lesions associated with VHL are asymptomatic initially, early diagnosis and the institution of an evidence-based surveillance protocol are of paramount importance. Screening, surveillance, and genetic counseling are key aspects in the management of patients diagnosed with VHL disease and often require a multidisciplinary approach and referral to specialized centers. This article will discuss the characteristic lesions seen with VHL disease, their diagnosis, screening protocols and management strategies, as well as an illustrative case to demonstrate the natural progression of the disease with classic imaging findings.

## Introduction

Von Hippel–Lindau disease (VHL) is an autosomal dominant and highly penetrant disorder that can present with a myriad of benign and malignant lesions involving various organ systems. With an annual incidence of one per 36,000 live births, it is inherited from an affected parent in 80% of the cases and arises *de novo* in the remaining 20% (1, 2). The mean age of initial tumor diagnosis is 26 years, and more than 90% of patients develop symptoms by the age of 65 (3). When hereditary, it arises due to a germline mutation in the VHL tumor suppressor gene located on the short arm of chromosome 3. As some of the lesions seen in this familial cancer syndrome are associated with significant morbidity and mortality, early detection and surveillance form the cornerstones of optimal management and can significantly improve life expectancy. Here, we discuss an interesting case of a young male who presented with several of the classical lesions characteristically seen in VHL.

## Case Report

**Figure 1:**
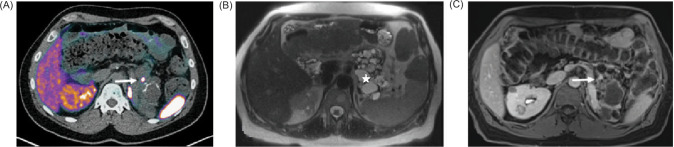
(A) Axial view on Gallium-68 Dotatate PET/CT demonstrates a focal uptake in the pancreatic tail (arrow); (B) Axial view on T-2 weighted HASTE sequence (Half Fourier Single-shot Turbo spin-Echo) demonstrates innumerable cysts within the pancreatic body and tail (star) consistent with the patient history of VHL; (C) Axial view on T-1 weighted VIBE post contrast sequence (Volumetric interpolated breath-hold examination) demonstrates multiple cysts and a focal enhancement in the pancreatic tail, corresponding to the focal radiotracer uptake, most consistent with a small neuroendocrine tumor (arrow).

**Figure 2:**
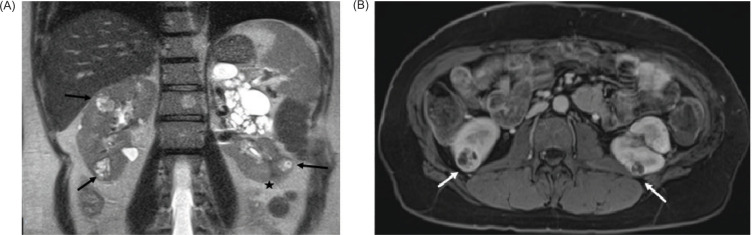
(A) Coronal view on T-2 weighted HASTE sequence demonstrates bilateral heterogeneous masses in the kidneys (arrows) and postsurgical scar in the lower pole of the left kidney (star); (B) Axial view on T-1 weighted VIBE post contrast sequence demonstrates heterogeneous enhancement of the renal masses consistent with renal cell carcinoma in this patient with VHL.

**Figure 3:**
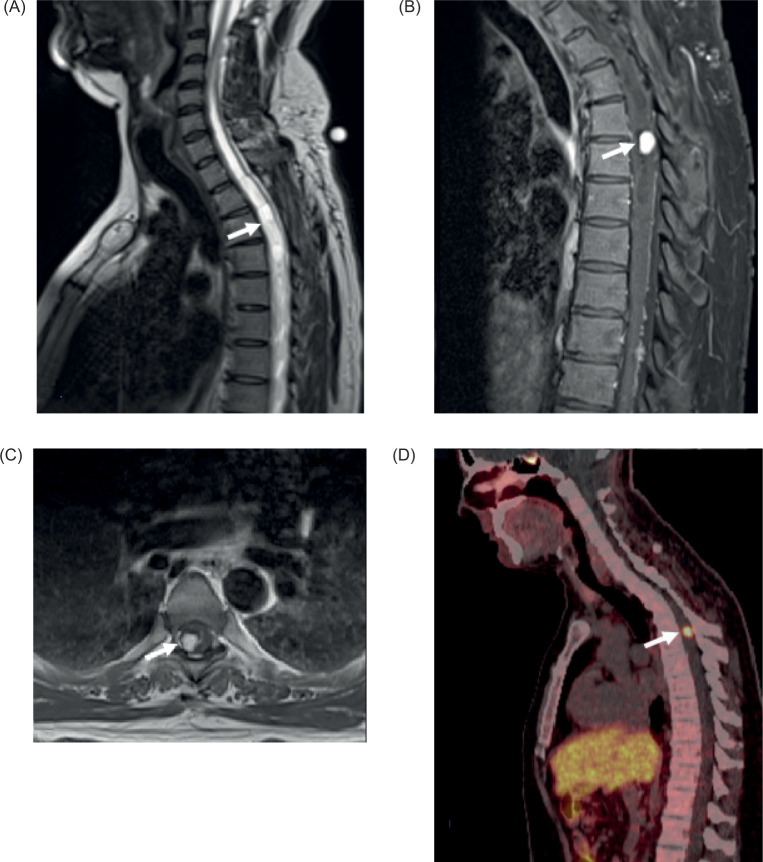
(A) Sagittal T2 image of the thoracic spine shows a long syringohydromyeliain of the cervicothoracic spine (arrow); (B) Sagittal T1 fat sat post contrast and (C) axial T1 post contrast demonstrates an avidly enhancing nodule within the thoracic spine, located within the syringohydromyelia and consistent with a hemangioblastoma in this patient with VHL disease (arrows); (D) Sagittal PET/CT Gallium 68 Dotatatedemonstrate avid uptake of the lesion (arrow).

**Figure 4:**
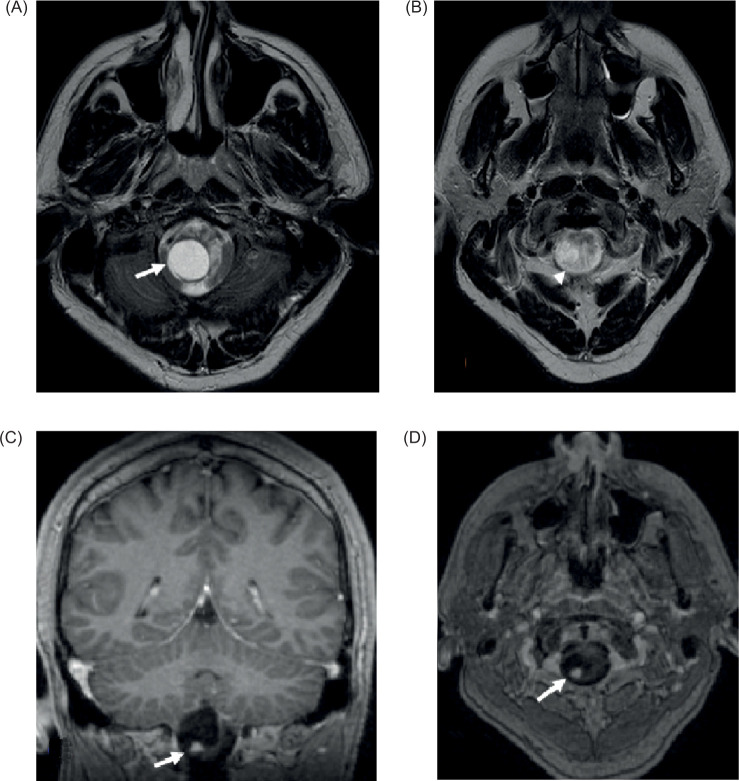
(A) and (B) Axial T2 images of the brain at the base of the skull level demonstrate a cystic signal lesion at the cervicomedullary junction [arrow in (A)] with peripheral more nodular area of intermediate signal [arrowhead in (B)]; (C) Coronal and (D) axial post contrast GRE demonstrates a cystic mass with peripheral enhancing nodule consistent with hemangioblastoma in this patient with VHL disease (arrows).

**Figure 5:**
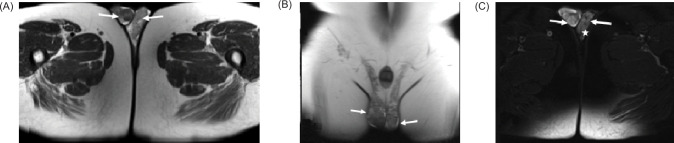
(A) Axial and (B) coronal HASTE images demonstrate lobulated scrotal, para-testicular masses showing predominantly intermediate signal (arrows); (C) Axial T2 fat sat image shows these lobulated masses (arrows), larger on the right, showing increase signal. Note the presence of enlarged left pampiniform plexus of veins consistent with varicocele (star).

**Figure 6:**
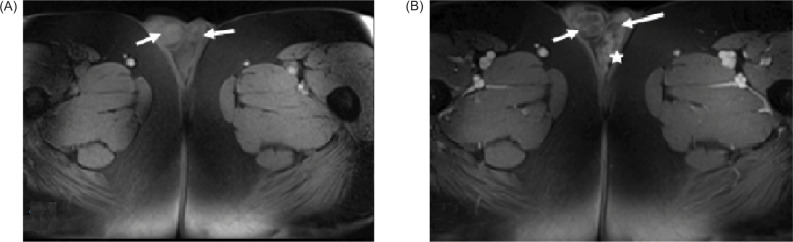
(A) Pre-and (B) post-contrast VIBE images show peripheral enhancement of the lesions. There is also tubular enhancement of the left scrotal veins (star). Note the mild increase signal of the lesions on pre-contrast VIBE suggestive of proteinaceous or hemorrhagic content (arrows).

This is the case of a 37-year-old male with a known history of VHL disease. The patient has a prior history of islet cell neuroendocrine tumor of the pancreas treated by Whipple surgery in 2003 (Figure 1), a history of left renal cell carcinoma, treated by partial left nephrectomy in 2004 (Figure 2), a history of thoracic spinal cord hemangioblastoma treated by resection in 2008 (Figure 3), and a more recent cerebellar hemangioblastoma resection in 2020 (Figure 4). The patient now presented with palpable scrotal masses bilaterally. Pelvic magnetic resonance imaging (MRI) demonstrated bilateral para-testicular masses, centered at the epidydimal heads, larger on the right (Figures 5 and 6). The masses are lobular in appearance, showing intermediate to high signal on T2-weighted images with only peripheral enhancement. The imaging characteristics are consistent with epidydimal cystadenoma. The key imaging findings of various lesions are summarized in Table 1.

**Table 1:** Summary of major imaging findings of different malignant lesions in the patient and their classic imaging findings.

Date	Imaging modality	Body region	Key findings	Diagnosis
2003	MRI	Abdomen	Multiple cysts and a focal enhancement in the pancreatic tail	Islet cell neuroendocrine tumor of the pancreas
	PET/CT		Focal uptake in the pancreatic tail	
2004	MRI	Abdomen	Bilateral heterogeneous masses in the kidneys on T2WI and heterogeneous enhancement on T1WI	Left renal cell carcinoma
2008	MRI	Vertebral column and spinal cord	Long syringohydromyeliain of the cervico-thoracic spine with avidly enhancing nodule within the thoracic spine	Spinalcord hemangioblastoma
	PET/CT		Avid uptake of the lesion	
2020	MRI	Head and neck	Cystic lesion at the cervicomedullary junction with peripheral nodular area of intermediate signal on T2WI	Cerebellar hemangioblastoma
2022	MRI	Scrotum	Lobulated paratesticular masses showing predominantly intermediate signal with Preand post-contrast VIBE images showing peripheral enhancement of the lesions	Epidydimal cystadenoma

CT, computed tomography; MRI, magnetic resonance imaging; PET, positron emission tomography.

## Discussion

The 232 aminoacid protein (pVHL) derived from the VHL gene (3p25.3) plays an essential role in the regulation of hypoxia-inducible factor (HIF)-1 and 2. The inactivation of this gene leads to hyperaccumulation of HIF, which leads to tumorigenesis and uncontrolled angiogenesis via activation of many hypoxia-inducible genes (4). Manifestations of VHL can be broadly classified as CNS lesions and visceral lesions (Table 2). CNS lesions include CNS hemangioblastomas, retinal hemangioblastomas, endolymphatic sac, and spinal cord tumors. Visceral lesions may be endocrine or non-endocrine. Pancreatic neuroendocrine proliferations, pheochromocytomas, and extra-adrenal paragangliomas are classical endocrine manifestations of VHL disease. Non-endocrine lesions include pancreatic and renal cysts, clear cell renal cell carcinoma (RCC), and epididymal and broad ligament cystadenomas. Tumors seen in VHL are usually multifocal, bilateral, and hypervascular in nature. Clinical classification of the disease is based on genotype–phenotype correlations (5). Type 1 disease is caused by partial or complete gene deletion or a nonsense mutation and is not associated with pheochromocytoma. In contrast, type 2 disease is caused by a missense mutation and is associated with the development of a pheochromocytoma. Type 2 disease is further classified as type 2A (low risk of renal cell carcinoma), type 2B (high risk of renal cell carcinoma), and type 2C (pheochromocytoma only) (5).

**Table 2:** Benign and malignant lesions seen in different organ systems in VHL spectrum with their MRI findings (20).

Organ system	Lesion	Imaging features
CNS	CNS hemangioblastomas: Seen in 70% of patients (range 60-80%) • Cerebellar (~60%; range 44–72%) • Spinal cord (~30%; range 13–50%) • Brainstem	T1WI: Hypointense mural nodule with CSF signal surrounding wall T2WI: Hyperintense mural nodule Nodule enhances with contrast while the wall does not (21)
	Choroid plexus papilloma	T1WI: Hypo-isointense to brain T2WI: Iso-hyperintense to brain Marked contrast enhancement (22)
Head and neck	Retinal hemangioblastoma Seen in 45–60% of the patients	T1WI: Hyperintense lesion T2WI: Hypo-isointense lesion Significant enhancement with contrast (23)
	Endolymphatic sac tumor Seen in 10–15% of the patients	T1WI: Focal hyperintensity T2WI: Heterogeneous signal intensity Heterogeneous enhancement with contrast (24)
Renal	Renal cell carcinoma Seen in 70% of the patients. Mostly clear cell type and bilateral	T1WI: Often has heterogeneous appearance T2WI: Hyperintense (clear cell carcinoma) or hypointense (papillary carcinoma) (25)
	Renal cysts Seen in 75% of the patients Often bilateral and multiple	Can be simple or complex cysts
	Renal angiomyolipoma	Non-fat-saturated sequence: High signal intensity Fat saturate sequence: loss of signal intensity (26)
Adrenal	Pheochromocytoma Seen in 25–30% of the patients	T1WI: Hypointense to adrenal T2WI: Mostly hyperintense to the adrenal Necrosis or hemorrhage might alter signal intensity Prolonged heterogeneous enhancement with contrast (27)
	Paraganglioma Seen in 15% of the patients	Extra-adrenal lesion. T1WI: Hypointense to adrenal T2WI: Hyperintense to adrenal Heterogenous prolonged enhancement with contrast (28)
Pancreas	Pancreatic cyst Seen in 40% of the patients	Can be unilocular, multilocular and may have a solid component
	Pancreatic neuroendocrine tumor Seen in 12.5% of the patients. Usually nonfunctional and multiple	Hypointense on T1WI and hyperintense on T2WI relative to pancreas. Restricted diffusion on DWI (29)
	Pancreatic adenocarcinoma	T1WI: Hypointense to pancreas T2WI: Variable Delayed enhancement compared to pancreas (30)
Liver	Liver cysts	T1WI: Homogeneous low signal intensity T2WI: Hyperintense to liver No contrast enhancement (31)
Urogenital	Epididymal cysts	May or may not have septations
	Epididymal cystadenoma	Isointense paratesticular masses (6)
	Broad ligament cystadenoma	Isointense mass-like lesion in adnexa (32)

CNS, central nervous system; CSF, cerebrospinal fluid; DWI, diffusion-weighted imaging.

Considered as a prototype tumor of VHL, CNS hemangioblastomamost commonly occurs in as seen in our patient. Other sites include spinal cord, brainstem, caudaequina, or supra-tentorial regions (6). Macroscopically, they are well-circumscribed solid masses with fluid-filled cysts. Histo-logically, they consist of stromal cells with lipid-laden cytoplasm and numerous vascular structures. Although benign, significant morbidity may occur due to rapid cyst expansion and peri-tumoral edema causing compression of adjacent structures. The gold standard for detection and monitoring of these lesions is contrast-enhanced MRI and the characteristic imaging finding is a well-defined homogenous cyst with an avidly enhancing nodule or solid component (6, 7). As seen in our patient, lesions in the spinal cord frequently coexist with cerebellar lesions. Therefore, once a cerebellar hemangioblastoma is identified, it is essential to image the entire spinal cord. In a study by Kanno et al., it was shown that once a patient was diagnosed with VHL, a spinal cord hemangioblastoma can be predicted to occur within 5 years (8). Asymptomatic lesions may be managed with close surveillance. For symptomatic lesions, microsurgical resection is the treatment of choice, and radiation may be used in patients who are not surgical candidates (9). Irrespective of the number of previous tumors and the presence or absence of symptoms, lifelong annual MRI scans of the brain and spinal cord are recommended (10). Retinal hemangioblastoma is the other CNS lesion seen very frequently in VHL patients and may lead to blindness in 15% of patients (9).

Screening with dilated fundoscopy and slit-lamp examination is recommended. Once detected, laser photocoagulation and cryotherapyare the mainstays of management (9).

Seen in more than two-thirds of VHL cases, multicentric renal cysts and clear cell RCCs are another significant cause of morbidity in these patients (11). The presence of bilateral or multifocal clear cell RCCs in patients younger than 50 years of age is an indication for VHL genetic screening (6). Often associated with lower-grade histology, VHL- related RCCs metastasize only when greater than 3 cm in diameter (9). Abdominal renal protocol CT scan is the most sensitive diagnostic modality. Considering the recurrent and multifocal nature of renal disease, surgical resection with partial nephrectomy or nephron-sparing surgery is recommended for tumors greater than 3 cm (12).

Two endocrine manifestations of VHL disease which warrant discussion are pancreatic neuroendocrine tumors and pheochromocytoma. Patients with VHL can develop pancreatic cysts, serous cystadenomas, and neuroendocrine tumors (NETs). Pancreatic lesions can be diagnosed with pre- and post-contrast abdominal CT and MR imaging (9). FDG PET may be used to detect NETs not visible on CT. NETs are usually positive for synaptophysin and chromogranin A on immunohistochemical analysis. Most of the NETs are nonfunctional and asymptomatic and, thus, are rare causes of significant morbidity or mortality. However, malignant conversion and metastases may lead to a poor prognosis. A conservative “watch-and-wait” strategy with annual imaging is appropriate for asymptomatic, small, and stable lesions. Surgical resection is preferred in presence of symptoms or features associated with an aggressive pattern of disease progression. These features include tumor size more than 3 cm, tumor doubling time less than 500 days, or a mutation in exon 3 (6). Pheochromocytomas in patients with VHL usually arise from the adrenal medulla and are bilateral and multifocal. Malignant transformation is rare. Diagnosis is made with biochemical testing for excess catecholamines and imaging. Laparoscopic surgery after appropriate preoperative medical management is the recommended treatment. Contact sports should be avoided if pancreatic or adrenal lesions are present (9). Epididymalcystadenoma, also seen in our patient, is another common manifestation involving the reproductive tract in young males with VHL disease. They are commonly bilateral and asymptomatic. Presenting as incidentally detected scrotal masses, they are benign tumors and typically managed conservatively (6).

Our case demonstrates how various benign and malignantlesions can develop over time in patients with VHL disease and underscores the importance of having knowledge of characteristic lesions, timely genetic testing, and periodic surveillance for follow-up. As most lesions associated with VHL are asymptomatic initially and follow a saltatory growth pattern with periods of quiescence (9), early diagnosis and the institution of an evidence-based surveillance protocol are of paramount importance. Diagnosis is based on clinical criteria, family history, and genetic testing. The presence of a single VHL-associated tumor is sufficient to make a clinical diagnosis in patients with a positive family history, while two such lesions are required to diagnose sporadic cases (2, 13). Whenever a clinical diagnosis is suspected, genetic screening should be performed using techniques such as Southern blotting, fluorescence *in situ* hybridization (FISH), or multiplex ligation-dependent probe amplification (MLPA) (14). Sequence variations detected in the VHL gene are then compared with the 377 known intragenic mutations associated with VHL disease (9). Screening, surveillance, and genetic counseling are key aspects in the management of patients diagnosed with VHL disease and often require a multidisciplinary approach and referral to specialized centers. After the diagnosis is made, initial screening workup includes an ophthalmologic exam including fundoscopy, MR imaging of the craniospinal axis, kidneys, pancreas, and liver, an audiology exam, and laboratory tests to detect pheochromocytoma. Various national surveillance guidelines for VHL are in place, and it is recommended that surveillance should start at birth and continue lifelong (13, 15). Surveillance includes clinical exams, imaging, and lab studies, and recommendations are age-dependent. From a radiological standpoint, it is generally recommended to do a baseline MRI with contrast of the CNS followed by repeat scans every 2 years. For abdominal lesions, MRI is the preferred modality and should be done every 2 years, however CT and US can be performed on case-to-case basis (13). These recommendations are only for organs without lesions. Once a malignancy is diagnosed in an organ, the imaging follow-up is personalized based on the lesion (13). Special considerations are required during pregnancy and prenatal and preimplantation genetic diagnosis should be provided for at-risk pregnancies.

Several pharmacological targets have been recently discovered that can help treat different lesions seen in VHL disease. Hypoxia-inducible factor subunit 2 alpha (HIF2α) inhibitorsis one such new category of drugs developed for VHL lesions. Belzutifan, a novel HIF2α inhibitor, recently received FDA approval for the management of nonmetastatic RCCs, pancreatic neuroendocrine tumors, and CNS hemangioblastomas in patients with VHL disease with *VHL* germline mutations (16, 17). Phase 1 and 2 studies have shown appropriate drug safety and tolerance with an overall response rate (ORR) of 49% and progression free survival of 96% at 4 months for localized RCC, ORR of 77% for pancreatic lesions, 30% for CNS hemangioblastomas and 100% for retinal hemangioblastomas. The drug also showed an ORR of 25% and disease control rate of 80% in metastatic RCC with a PFD of 16.5 months. Recent evidence has also shown that inhibiting VEGF–VEGFR signaling can alter the tumor microenvironment of RCC such that the tumor becomes more immune-responsive (18, 19). This has led to the use of combination of immune checkpoint inhibitors (PD-1/PDL-1 inhibitors and CTLA-4 inhibitors) in treatment of VHL. Other drug categories suchanti-VEGFR and tyrosine kinase inhibitors are also being actively investigated for the treatment of VHL lesions. Ongoing clinical trials and future studies will reveal the efficacy and safety of the use of different combinations of drugs in the treatment of VHL.

## Conclusion

In conclusion, VHL is a hereditary, multisystem cancer syndrome caused by the inactivation of the VHL tumor suppressor gene on chromosome 3. Familiarity with the clinical, pathophysiological, and imaging characteristics of various associated lesions is important for early detection. Identification of lesions pathognomonic of the disease or a positive family history should prompt genetic screening for VHL gene mutations. Once detected, a multidisciplinary approach tailored to factors such as patient age, lesions, and comorbidities is essential for improving both life expectancy and quality of life. Screening, surveillance, and genetic counseling are key features of optimal management of the disease, and a proper protocol must be put in place for all diagnosed cases.
